# Heavier smoking may lead to a relative increase in waist circumference: evidence for a causal relationship from a Mendelian randomisation meta-analysis. The CARTA consortium

**DOI:** 10.1136/bmjopen-2015-008808

**Published:** 2015-08-11

**Authors:** Richard W Morris, Amy E Taylor, Meg E Fluharty, Johan H Bjørngaard, Bjørn Olav Åsvold, Maiken Elvestad Gabrielsen, Archie Campbell, Riccardo Marioni, Meena Kumari, Tellervo Korhonen, Satu Männistö, Pedro Marques-Vidal, Marika Kaakinen, Alana Cavadino, Iris Postmus, Lise Lotte N Husemoen, Tea Skaaby, Tarun Veer Singh Ahluwalia, Jorien L Treur, Gonneke Willemsen, Caroline Dale, S Goya Wannamethee, Jari Lahti, Aarno Palotie, Katri Räikkönen, Alex McConnachie, Sandosh Padmanabhan, Andrew Wong, Christine Dalgård, Lavinia Paternoster, Yoav Ben-Shlomo, Jessica Tyrrell, John Horwood, David M Fergusson, Martin A Kennedy, Ellen A Nohr, Lene Christiansen, Kirsten Ohm Kyvik, Diana Kuh, Graham Watt, Johan G Eriksson, Peter H Whincup, Jacqueline M Vink, Dorret I Boomsma, George Davey Smith, Debbie Lawlor, Allan Linneberg, Ian Ford, J Wouter Jukema, Chris Power, Elina Hyppönen, Marjo-Riitta Jarvelin, Martin Preisig, Katja Borodulin, Jaakko Kaprio, Mika Kivimaki, Blair H Smith, Caroline Hayward, Pål R Romundstad, Thorkild I A Sørensen, Marcus R Munafò, Naveed Sattar

**Affiliations:** 1 School of Social and Community Medicine, University of Bristol, Bristol, UK; 2 Department of Primary Care and Population Health, UCL, London, UK; 3 MRC Integrative Epidemiology Unit (IEU) at the University of Bristol, University of Bristol, Bristol, UK; 4 UK Centre for Tobacco and Alcohol Studies and School of Experimental Psychology, University of Bristol, Bristol, UK; 5 Department of Public Health, Faculty of Medicine, Norwegian University of Science and Technology, Trondheim, Norway; 6 Forensic Department, Research Centre Bröset, St Olav's University Hospital Trondheim, Trondheim, Norway; 7 Department of Endocrinology, St. Olavs Hospital, Trondheim University Hospital, Trondheim, Norway; 8 Department of Laboratory Medicine, Children's and Women's Health, The Faculty of Medicine, Norwegian University of Science and Technology, Trondheim, Norway; 9 Medical Genetics Section, Centre for Genomic and Experimental Medicine, Institute of Genetics and Molecular Medicine, University of Edinburgh, Edinburgh, UK; 10 Centre for Cognitive Ageing and Cognitive Epidemiology, University of Edinburgh, Edinburgh, UK; 11 Queensland Brain Institute, University of Queensland, Brisbane, Queensland, Australia; 12 Institute for Social and Economic Research, University of Essex, Colchester, UK; 13 Department of Public Health, Hjelt Institute, University of Helsinki, Helsinki, Finland; 14 Institute of Public Health and Clinical Nutrition, University of Eastern Finland, Kuopio, Finland; 15 National Institute for Health and Welfare, Helsinki, Finland; 16 Department of Internal Medicine, Lausanne University Hospital, Lausanne, Switzerland; 17 Institute of Health Sciences, University of Oulu, Oulu, Finland; 18 Biocenter Oulu, University of Oulu, Oulu, Finland; 19 Centre for Environmental and Preventive Medicine, Wolfson Institute of Preventive Medicine, Barts and the London School of Medicine and Dentistry, Queen Mary University of London, London, UK; 20 Population, Policy and Practice, UCL Institute of Child Health, University College London, UK; 21 Department of Gerontology and Geriatrics, Leiden University Medical Center, Leiden, The Netherlands; 22 Netherlands Consortium of Healthy Ageing, Leiden, The Netherlands; 23 Research Centre for Prevention and Health, the Capital Region of Denmark, Denmark; 24 Novo Nordisk Foundation Center for Basic Metabolic Research, Section of Metabolic Genetics, Faculty of Health and Medical Sciences, University of Copenhagen, Copenhagen, Denmark; 25 Steno Diabetes Center, Gentofte, Denmark; 26 COPSAC, Copenhagen Prospective Studies on Asthma in Childhood, Herlev and Gentofte Hospital, University of Copenhagen, Copenhagen, Denmark; 27 Department of Biological Psychology, Netherlands Twin Register, VU University, Amsterdam, The Netherlands; 28 Faculty of Epidemiology and Population Health, London School of Hygiene & Tropical Medicine, London, UK; 29 Institute of Behavioural Sciences, University of Helsinki, Helsinki, Finland; 30 Folkhälsan Research Centre, Helsinki, Finland; 31 Wellcome Trust Sanger Institute, Wellcome Trust Genome Campus, Cambridge, UK; 32 Institute for Molecular Medicine Finland (FIMM), University of Helsinki, Finland; 33 The Medical and Population Genomics Program, The Broad Institute of MIT and Harvard, Cambridge, Massachusetts, USA; 34 Robertson Centre for Biostatistics, University of Glasgow, Glasgow, UK; 35 Institute of Cardiovascular and Medical Sciences, University of Glasgow, Glasgow, UK; 36 MRC Unit for Lifelong Health and Ageing at UCL, London, UK; 37 Department of Environmental Medicine, Institute of Public Health, University of Southern Denmark, Odense, Denmark; 38 European Centre for Environment and Human Health, University of Exeter Medical School, Truro, UK; 39 Genetics of Complex Traits, University of Exeter Medical School, Exeter, UK; 40 Department of Psychological Medicine, University of Otago, Christchurch, New Zealand; 41 Department of Pathology, University of Otago, Christchurch, New Zealand; 42 Institute for Clinical Research, University of Southern Denmark, Odense, Denmark; 43 Department of Epidemiology, Biostatistics and Biodemography, Institute of Public Health, University of Southern Denmark, Denmark; 44 University of Glasgow, Glasgow, UK; 45 Department of General Practice and Primary Health Care, University of Helsinki, Helsinki, Finland; 46 Unit of General Practice, Helsinki University Central Hospital, Helsinki, Finland; 47 Vasa Central Hospital, Vasa, Finland; 48 Population Health Research Institute, St George's University of London, London, UK; 49 Department of Clinical Experimental Research, Glostrup University Hospital, Glostrup, Denmark.; 50 Department of Clinical Medicine, Faculty of Health and Medical Sciences, University of Copenhagen, Copenhagen, Denmark; 51 Department of Cardiology, Leiden University Medical Center, Leiden, The Netherlands; 52 Durrer Center for Cardiogenetic Research, Amsterdam, The Netherlands; 53 Interuniversity Cardiology Institute of the Netherlands, Utrecht, The Netherlands; 54 Centre for Population Health Research, School of Health Sciences and Sansom Institute of Health Research, University of South Australia, Adelaide, Australia; 55 South Australian Health and Medical Research Institute, Adelaide, South Australia, Australia; 56 Unit of Primary Care, Oulu University Hospital, Oulu, Finland; 57 Department of Children and Young People and Families, National Institute for Health and Welfare, Oulu, Finland; 58 Department of Epidemiology and Biostatistics, MRC Health Protection Agency (HPA) Centre for Environment and Health, School of Public Health, Imperial College London, London, UK; 59 Department of Psychiatry, Lausanne University Hospital, Lausanne, Switzerland; 60 Institute for Molecular Medicine Finland FIMM, University of Helsinki, Helsinki, Finland; 61 Department of Epidemiology and Public Health, University College London, London, UK; 62 Division of Population Health Sciences, University of Dundee, Ninewells Hospital and Medical School, Dundee, UK; 63 Medical Research Council Human Genetics Unit, Institute of Genetics and Molecular Medicine, University of Edinburgh, Edinburgh, UK; 64 Institute of Preventive Medicine, Bispebjerg and Frederikberg Hospitals, The Capital Region, Copenhagen, Denmark; 65 Faculty of Medicine, BHF Glasgow Cardiovascular Research Centre, Glasgow, UK

**Keywords:** EPIDEMIOLOGY, GENETICS

## Abstract

**Objectives:**

To investigate, using a Mendelian randomisation approach, whether heavier smoking is associated with a range of regional adiposity phenotypes, in particular those related to abdominal adiposity.

**Design:**

Mendelian randomisation meta-analyses using a genetic variant (rs16969968/rs1051730 in the *CHRNA5-CHRNA3-CHRNB4* gene region) as a proxy for smoking heaviness, of the associations of smoking heaviness with a range of adiposity phenotypes.

**Participants:**

148 731 current, former and never-smokers of European ancestry aged ≥16 years from 29 studies in the consortium for Causal Analysis Research in Tobacco and Alcohol (CARTA).

**Primary outcome measures:**

Waist and hip circumferences, and waist-hip ratio.

**Results:**

The data included up to 66 809 never-smokers, 43 009 former smokers and 38 913 current daily cigarette smokers. Among current smokers, for each extra minor allele, the geometric mean was lower for waist circumference by −0.40% (95% CI −0.57% to −0.22%), with effects on hip circumference, waist-hip ratio and body mass index (BMI) being −0.31% (95% CI −0.42% to −0.19), −0.08% (−0.19% to 0.03%) and −0.74% (−0.96% to −0.51%), respectively. In contrast, among never-smokers, these effects were higher by 0.23% (0.09% to 0.36%), 0.17% (0.08% to 0.26%), 0.07% (−0.01% to 0.15%) and 0.35% (0.18% to 0.52%), respectively. When adjusting the three central adiposity measures for BMI, the effects among current smokers changed direction and were higher by 0.14% (0.05% to 0.22%) for waist circumference, 0.02% (−0.05% to 0.08%) for hip circumference and 0.10% (0.02% to 0.19%) for waist-hip ratio, for each extra minor allele.

**Conclusions:**

For a given BMI, a gene variant associated with increased cigarette consumption was associated with increased waist circumference. Smoking in an effort to control weight may lead to accumulation of central adiposity.

**Strengths and limitations of this study**

- This is a very large Mendelian randomisation study of the relationship between smoking and several anthropometric phenotypes relating to regional adiposity.
- Data included never, former and current smokers from a very wide spectrum of ages among 29 studies.
- By using a genetic variant associated with smoking heaviness as a proxy for smoking heaviness, bias from confounding is minimised and findings are not affected by reverse causality.
- Data for direct measures of fat, such as fat mass, and the biomarker leptin were available for only about one fifth of the participants whose weight, height, waist and hip were measured.
- Participants were exclusively of self-reported European ancestry, and were mostly recruited in European countries.

## Introduction

Tobacco is the single most important cause of preventable death globally: one in two young people taking up lifelong cigarette smoking will die of causes related to it.^1^ Enormous efforts have gone into developing interventions for smoking cessation. Spontaneous cessation rates are low due to the high proportion of smokers who are dependent on nicotine, and effective treatments are still not widely available. One barrier to smoking cessation is the fear of weight gain. In a study of almost 2000 smokers in the USA, recruited into a trial of bupropion and/or nicotine inhalers to promote cessation, 50% of female and 26% of male smokers reported that gaining weight discouraged them from trying to quit,^2^ while among adults in Finland, daily smokers were found to report more weight concerns than former smokers or occasional smokers.^3^

A genetic variant in the chromosome 15 *CHRNA5-CHRNA3-CHRNB4* gene region (rs16969968) codes for a functional amino acid change D398N in the nicotinic receptor α5 subunit. The SNP rs16969968, which is in perfect linkage disequilibrium with SNP rs1051730 in European populations, is associated with smoking quantity among smokers.^4^ The minor allele of this variant is associated with an average increase in smoking amount of one cigarette per day in smokers and increases in cotinine (a metabolite of nicotine) levels.^5^ ^6^ It has also been found that the variant was associated with a lower mean body mass index (BMI),^7–9^ thus adding evidence that heavier smoking leads to lower BMI. The latter study also noted lower waist and hip circumferences among smokers with the variant.^8^ However, prior observational evidence suggests that waist circumference and waist-hip ratio may be higher in smokers than in non-smokers after adjusting for BMI.^10^ It has also been observed that smoking in adolescence predicts abdominal obesity in adulthood.^11^ Moreover, heavy smokers exhibit greater central adiposity than light smokers, based on an analysis of middle-aged smokers of European ancestry.^12^ These studies suggest that smoking leads to a central fat accumulation at the expense of peripheral fat loss, particularly in women.^13^ In addition, there are also suggestions that smoking may lead to loss of muscle mass as indicated by lower hip circumferences in smokers. This is of high public health relevance in view of the reportedly greater impact of increased central adiposity both on mortality^14^ ^15^ and on the development of diabetes, especially among women,^16^ ^17^ and since smoking is associated with an increased risk of type 2 diabetes.^18^

We previously used Mendelian randomisation methods to investigate the effect of smoking quantity on BMI.^7^ ^9^ This method exploits Mendel's laws concerning the random assortment of alleles at the time of gamete formation so that individuals are allocated at random to having 0, 1 or 2 alleles in the rs1051730/rs16969968 genotype. The effect of this genotype on smoking quantity among smokers has been demonstrated,^6^ and thus the inverse relationship between allele count and BMI is not subject to effects of confounding and reverse causality. Using a substantial pool of studies in the consortium for Causal Analysis Research in Tobacco and Alcohol (CARTA), we have extended our use of Mendelian randomisation methods to examine the effect of smoking quantity on a range of adiposity phenotypes. We test the hypotheses that (1) phenotypes representing central adiposity are affected by smoking quantity differentially from other phenotypes, and (2) these effects are more marked among women than among men.

## Methods

### Study populations

We used data on individuals (≥16 years) of self-reported European ancestry from 29 studies from the CARTA consortium (http://www.bris.ac.uk/expsych/research/brain/targ/research/collaborations/carta/): the 1958 Birth Cohort (1958BC), the Avon Longitudinal Study of Parents and Children (ALSPAC, including both mothers and children), the British Regional Heart Study (BRHS), the British Women's Heart and Health Study (BWHHS), the Caerphilly Prospective Study (CaPS), the Christchurch Health and Development Study (CHDS), CoLaus, the Danish Monica study (Dan-MONICA), the Exeter Family Study of Child Health (EFSOCH), the English Longitudinal Study of Ageing (ELSA), the National FINRISK studies, GEMINAKAR, GS:SFHS (Generation Scotland: Scottish Family Health Study), the Genomics of Overweight Young Adults (GOYA) females, GOYA males, the Helsinki Birth Cohort Study (HBCS), Health2006, Health2008, the Nord-Trøndelag Health Study (HUNT), Inter99, MIDSPAN, the Northern Finland Birth Cohorts (NFBC 1966 and NFBC 1986), the National Health and Nutrition Examination Survey (NHANES), the MRC National Survey of Health & Development (NSHD), the Netherlands Twin Register (NTR), the PROspective Study of Pravastatin in the Elderly at Risk (PROSPER) and Whitehall II. All studies received ethics approval from the local research ethics committees. Further details of these studies are provided in online supplementary material.

### Genotype

Within each study, individuals were genotyped for one of two single nucleotide polymorphisms (SNPs) in the *CHRNA5-A3-B4* nicotinic receptor subunit gene cluster, either rs16969968 or rs1051730. These SNPs are in perfect linkage disequilibrium with each other in Europeans (R^2^=1.00 in HapMap 3, http://hapmap.ncbi.nlm.nih.gov/) and therefore represent the same genetic signal. Where studies had data available for both SNPs, we used the SNP that was genotyped in the largest number of individuals. Details of genotyping methods within each study are provided in online supplementary material.

### Adiposity measures

Direct physical measurements included weight, height, waist and hip circumferences, arm circumference, triceps skinfold and subscapular skinfold thickness. Fat mass and fat-free mass were available from bioimpedance measures, while leptin and adiponectin were the two biochemical markers related to fat mass.

BMI (weight/height^2^) and waist-hip ratio (waist/hip) were calculated.

Waist circumference and waist-hip ratio were taken as key measures of central adiposity, while BMI acted as a non-specific measure of adiposity for purposes of adjustment in regression analysis.

### Smoking status

Smoking status was self-reported (either by questionnaire or interview) at the same time as regional adiposity measures for all studies, with the exception of 1958 BC (see online supplementary material). Individuals were classified as current, former, ever (ie, current and former combined) or never cigarette smokers. Where information on pipe and cigar smoking was available, individuals reporting being current or former smokers of pipes or cigars but not cigarettes were excluded from all analyses.

For studies with adolescent populations (ALSPAC children and NFBC 1986), analyses were restricted to current daily smokers who reported smoking at least one cigarette per day (current smokers) and individuals who had never tried smoking (never-smokers).

### Statistical analysis

Analyses were conducted within each contributing study using Stata (Stata Corp, College Station, Texas, USA) and R (R Foundation for Statistical Computing, Vienna, Austria. http://www.R-project.org) software, following the same analysis plan. Analyses were restricted to individuals with full data on smoking status and rs1051730/rs16969968 genotype, and having data on at least one of the regional adiposity phenotypes.

Within each study, genotype frequencies were tested for deviation from the Hardy Weinberg Equilibrium (HWE) using a χ^2^ test. Mendelian randomisation analyses of the association between rs1051730/rs16969968 and each regional adiposity phenotype were performed using linear regression, stratified by smoking status (never, former and current) and sex, and adjusted for age. Apart from height, natural logarithmic transforms were taken of every anthropometric phenotype. An additive genetic model was assumed on log values, so that each effect size could be exponentiated to represent the percentage increase per minor (risk) allele. These analyses were presented separately for each smoking status category. All phenotypic measures were further adjusted for log(BMI) (apart from weight, height and BMI itself), thus assessing the effect of the particular adiposity measure after adjusting for this global weight measure. Log(weight) was adjusted for height instead of log(BMI). Since adjustment for ratio variables in anthropometric studies has been criticised,^19^ we further adjusted waist circumference for log(weight) and height. Finally, we repeated analysis of waist circumference adjusted for BMI restricted to participants with BMI under 30 kg/m^2^; 95% CIs have been quoted for all effect sizes.

Meta-analysis was also carried out of the relationship between reported daily cigarette consumption and rs1051730/rs16969968 genotype, among current smokers.

Although analyses were carried out separately for males and females, the estimates were combined where no evidence for separate sex effects was seen. For NHANES, which has a survey design, Taylor series linearisation was implemented to estimate variances. For studies including related family members, appropriate methods were used to adjust SEs: in GEMINAKAR, twin pair identity was included as a cluster variable in the model; in MIDSPAN, linear mixed effects regression models fitted using restricted maximum likelihood were used to account for related individuals, while in NTR, only unrelated individuals were included. ALSPAC mothers and children were analysed as separate samples; as there are related individuals across these samples, sensitivity analyses were performed excluding each of these studies in turn.

Results from individual studies were meta-analysed in Stata (V.13) using the ‘metan’ command from Stata. Where there was evidence of heterogeneity between studies (I^2^ >50%), it was planned that both fixed and random effects analyses would be performed: however, as this never occurred, results for fixed effects analysis only are shown. Meta-regression analysis, using the ‘metareg’ command from Stata, was used to examine whether SNP effects varied by smoking status or by sex, or by a smoking by sex combination.

## Results

### Descriptive statistics

The maximum sample size available, with genotype recorded, was 148 731 for weight, height and BMI over 29 studies. The data on individuals with weight, height, smoking status and genotype recorded included 66 809 never-smokers, 43 009 former smokers and 38 913 current smokers. Waist circumference was available in 28 studies (n=142 381), and hip circumference and waist-hip ratio in 25 studies (n=139 667). Measures of fat mass and fat-free mass were provided by 10 studies (n=28 231), arm circumference by nine studies (n=72 536), and skinfolds by five studies (n=7758). Finally, leptin and adiponectin were measured in nine studies (n=23 630 and 19 191, respectively). Overall, 47% of the combined study population was male. The median age within the contributing studies ranged from 16–74 years. Descriptive statistics for each of the study populations are found in the supplementary material (see online supplementary table S1).

Minor allele frequency for rs1051730/rs16969968 ranged between 0.31 and 0.36. There was no strong evidence for deviation from the Hardy-Weinberg Equilibrium in any of the studies (p values all ≥0.09, see online supplementary table S2).

### Mendelian randomisation analysis

Table 1 shows the per-allele increases in each phenotype within each smoking status category. As previously shown,^9^ the increase in BMI was positive in never-smokers: +0.35% (95% CI 0.18% to 0.52%; p=6.38×10^−5^), non-significant in former smokers: −0.14% (95% CI −0.34% to +0.07%; p=0.19) and significantly inverse in current smokers: −0.74% (95% CI −0.96% to −0.51%; p=2×10^−10^). The full results for each contributing study are shown in online supplementary figure S1.

**Table 1:** Per allele percentage increases in measures of regional adiposity (BMI, weigh, waist circumference, hip circumference, waist-hip ratio) among never, ex and current smokers, before and after adjustment for BMI

		Adjusted for age				Adjusted for age and BMI		p For interaction*
BMI (kg/m^2^)	Never-smokers	Former smokers	Current smokers	p For interaction*	Never-smokers	Former smokers	Current smokers	
% increase	0.35	−0.14	−0.74		–			
95% CI	(0.18 to 0.52)	(−0.34 to 0.07)	(−0.96 to −0.51)					
p	6.38×10^−5^	0.19	2.00×10^−10^	4.95×10^−13^				
N	66 809	43 009	38 912					
I^2^	14%	0%	0%					
Waist circumference (cm)								
% increase	0.23	−0.07	−0.40		0.01	0.06	0.14	
95% CI	(0.09 to 0.36)	(−0.24 to 0.09)	(−0.57 to −0.22)		(−0.06 to 0.08)	(−0.02 to 0.15)	(0.05 to 0.22)	
p	0.0012	0.37	1.69×10^−5^	3.85×10^−7^	0.72	0.15	0.003	0.087
N	64 265	40 756	37 360					
I^2^	14%	0%	10%		0%	0%	13%	
Hip circumference (cm)								
% increase	0.17	−0.07	−0.31		0.02	0.02	0.02	
95% CI	(0.08 to 0.26)	(−0.17 to 0.04)	(−0.42 to −0.19)		(−0.03 to 0.07)	(−0.04 to 0.08)	(−0.05 to 0.08)	
p	2.95×10^−4^	0.23	2.55×10^−7^	1.79×10^−9^	0.38	0.54	0.59	0.99
N	62 323	40 512	36 833					
I^2^	7%	0%	0%		16%	0%	0%	
Waist-hip ratio								
% increase	0.07	0	−0.08		−0.01	0.04	0.1	
95% CI	(−0.01 to 0.15)	(−0.10 to 0.10)	(−0.19 to 0.03)		(−0.08 to 0.06)	(−0.04 to 0.13)	(0.02 to 0.19)	
p	0.087	0.97	0.14	0.083	0.78	0.30	0.02	0.13
N	62 322	40 512	36 833					
I^2^	21%	9%	15%		0%	0%	13%	

*Interaction assessed by assessing heterogeneity between effect estimates according to smoking status, with a fixed effects model.

BMI, body mass index.

The waist circumference was higher per minor allele in never-smokers: +0.23% (95% CI 0.09% to 0.36%; p=0.0012), non-significantly related in former smokers −0.07% (95% CI −0.24% to 0.09%; p=0.37) and lower in current smokers −0.40% (95% CI −0.57 to −0.22 p=1.69×10^−5^): differences among smoking groups were highly significant (p=3.85×10^−7^; see online supplementary figure S2. The per-allele effect on waist circumference in current smokers was about half the magnitude of that seen for BMI. After adjustment for log(BMI), the minor allele of rs1051730-rs16969968 was not associated with waist circumference in either never-smokers: +0.01% (95% CI −0.06 to 0.08; p=0.72) or former smokers +0.06% (95% CI −0.02% to 0.15%; p=0.15). However, in current smokers, the minor allele was associated with a 0.14% (95% CI 0.05% to 0.22%; p=0.003) higher waist circumference after adjustment for log(BMI). Very similar results were seen in all three smoking status categories after waist was adjusted for log(weight) and height instead of for log(BMI). Effects of genotype on waist circumference were shown to differ between smoking status categories before adjustment (p=3.85×10^−7^) but only weakly after adjustment for log(BMI) (p=0.102), and after adjustment for log(weight) and height (p=0.018). Little heterogeneity of study results was evident (I^2^≤25% within all smoking groups). After restricting analysis to participants with BMI under 30 kg/m^2^, we found that the percentage increases in waist circumference (after adjustment for log(BMI)) were 0.04% (95% CI −0.03% to 0.12%) for never-smokers, 0.03% (95% CI −0.06% to 0.13%) for ex-smokers and 0.12% (95% CI 0.02% to 0.21%) for current smokers: however, the test for difference in effects gave p=0.41.

Unadjusted results for hip circumference were very similar to that seen for waist, both in direction and magnitude, in all smoking status groups (see online supplementary figure S3). However, after adjustment for log(BMI), effects were not apparent in any of the three groups, and nor was the interaction of gene and smoking status.

Results for the waist-hip ratio were similar to the BMI, waist and hip circumferences in direction but were smaller in magnitude: +0.07%, 0.00% and −0.08% increases in never-smokers, former smokers and current smokers, respectively (p=0.083 for differences between smoking categories; see online supplementary figure S4). After adjustment for log(BMI), increases remained non-significant for never-smokers and former smokers (−0.01% and 0.04%) but increased significantly among current smokers (0.10%) (p=0.13 for differences among smoking groups).

For several other phenotypes, per-allele decreases were observed in current smokers that exceeded those seen either in former or never-smokers (see online supplementary table S4). However, there was only statistical evidence for decreases among current smokers for arm circumference (p=8.4×10^−5^) and leptin (p=0.025), while the difference between smoking groups was only significant for arm circumference (p=3.29×10^−4^). Both effects became non-significant after adjustment for log(BMI). Fat mass and fat-free mass, after adjustment by height, showed differences in effects by smoking group. These effects were more due to per-allele increases seen among never-smokers than decreases among current smokers.

Meta-regression analyses showed no clear evidence for associations between genotype and each adiposity phenotype being modified by sex: p values exceeded 0.1 for all phenotypes, adjusted or unadjusted, apart from hip circumference. The per-allele decreases in hip circumference among current smokers appeared more marked among women (p=0.067), but this effect was no longer apparent after adjusting for BMI (p=0.51).

The mean difference in daily cigarette consumption was 0.77 among current smokers (95% CI 0.67 to 0.88, I^2^=17%).

## Discussion

This meta-analysis of 29 studies comprising almost 150 000 participants with key adiposity phenotypes has demonstrated, first, that a variant associated with increased cigarette consumption was associated not only with lower BMI among current smokers, consistent with earlier findings,^7^ ^8^ but also with lower waist and hip circumferences. Second, the inverse association of the variant with lower waist circumference among current smokers changed direction after adjusting for BMI. The variant was positively associated with waist circumference but associated neither with hip circumference after BMI adjustment nor waist-hip ratio. Our results suggest that for every copy of the minor allele associated with cigarette consumption (ie, increasing cigarette per day consumption by approximately one cigarette), waist circumference will be increased by 0.14% if BMI were to remain constant. This suggests a preferential redistribution towards central adiposity associated with higher cigarette consumption: this important finding is in keeping with our hypothesis and extends current observational data.

We also observed that none of the effects were modified by sex, contrary to our second hypothesis. Finally, we have already noted among never-smokers an unexpected positive association of the gene variant with BMI^9^: the current analysis demonstrates this same association with waist and hip circumferences. This occurred in the opposite direction to the inverse association of various adiposity measures with the gene variant seen in current smokers (before adjustment for BMI).

The analysis consisted of never, former and current smokers from a very wide spectrum of ages among the 29 studies. The sample size was very large for the primary phenotypes considered here. Participants were exclusively of self-reported European ancestry, and were mostly recruited in European countries. Data for direct measures of fat, such as fat mass, and the biomarker leptin were available for only about one-fifth of the participants whose weight, height, waist and hip were measured. Effects according to genotype for these phenotypes showed broadly similar results for the three smoking categories to those seen for BMI.

Mendelian randomisation has proved a powerful tool for eliciting causal associations between phenotypic measures.^20^ In the present analysis, Mendel's laws concerning random assignment of genotype should produce an unconfounded comparison between the genotype influencing smoking consumption and the outcomes of interest, namely anthropometric phenotypes. Furthermore, since this random assignment occurs at the very outset of life, the associations between genotype and anthropometric measures cannot be due to reverse causality. If the genotype only influences smoking consumption, and not the initiation of smoking, then the relationship between genotype and anthropometric outcomes would only be expected among smokers.

In fact, while the variant was associated with lower waist and hip circumferences among current smokers, it was associated with greater waist and hip circumferences among never-smokers. This suggests that the true effect among current smokers may be even greater than estimated. When we adjusted waist circumference for BMI, there was no association with the gene variant among never-smokers. The relative proportions of ever-smokers and never-smokers were not clearly associated with genotype in the CARTA consortium, as reported elsewhere.^9^

The reversal of the association between waist circumference and allele count from negative to positive among current smokers after adjustment for BMI may be consistent with alternative explanations. First, heavy smokers may have less muscle mass; however, no association between allele count and fat-free mass could be detected in our analysis among smokers. Second, the test for interaction for smoking status and allele count on waist circumference after adjustment was of weak statistical significance. Third, the adjustment of one measure of adiposity with another with which it is highly correlated may have caused a spurious association. We repeated our analysis for participants with BMI under 30 only, where the correlation was more modest, and obtained similar results, albeit with reduced evidence for an effect.

Stratification of our analyses by smoking status could, in theory, introduce bias by conditioning on a collider (rs1051730/rs16969968).^21^ This variant shows some evidence for association with smoking cessation (current vs former smoking).^22^ While this is a possibility, no effect modifications of this variant with potential confounders by smoking status were demonstrated among 56 625 participants in the HUNT study.^8^

Cross-sectional observational data from Switzerland has demonstrated that waist and hip circumferences were more strongly related to the number of cigarettes smoked per day than was BMI,^13^ while in Scotland being a smoker was associated with greater central adiposity among women.^12^ In a Finnish longitudinal twin cohort study, smoking in adolescence predicted abdominal obesity in adulthood.^11^ Observational data are, however, prone to confounding and reverse causality, and the present study adds some evidence that the associations reported are likely to be causal.

Some observational studies have noted that low fat-free mass^23^ and bone mineral density^24^ were more common among smokers. The present analysis has not substantiated the association with fat-free mass, although our sample size was much more limited for this phenotype.

Our findings resonate with observational studies which have shown associations between smoking and risk of diabetes,^17^ ^18^ especially as analysis of the British Women's Heart and Health Study showed that abdominal adiposity was a stronger predictor of diabetes than was BMI.^16^ Waist circumference and waist-to-hip ratio were strongly associated, independently of BMI, with the risk of death among 359 387 participants from nine countries in the European Prospective Investigation into Cancer and Nutrition.^15^ Therefore, the health hazards of smoking could well be enhanced or partly mediated through increasing abdominal adiposity. In addition, the desire of many smokers to use smoking as a means of weight control^2^ might be counterproductive if a loss of weight is accompanied by a relative increase in waist circumference: this possibility could be used in counselling people seeking to quit smoking.

People who quit smoking appear to be at increased risk of acquiring diabetes in the short term but this was not explained by weight gain in a Japanese population.^25^ This study took place almost exclusively among white European participants, and replication of the findings among other ethnic populations would be of great value. This is especially urgent on a global scale since smoking levels are increasing among several non-white ethnic groups, and this is seen to be partly responsible for increases in coronary heart disease mortality in Beijing, China,^26^ in Syria^27^ and in Tunisia among women.^28^ In addition, increases in average waist circumference have been observed even when average BMI levels have remained constant,^29^ and metabolic disorders, especially diabetes, have increased in prevalence.^30^ It is thus possible that increased CHD mortality will be partly fuelled by increasing smoking levels.

Mendelian randomisation studies have more potential than traditional observational epidemiological studies to establish causality for specific exposures,^20^ and they should now be used to investigate other impacts of smoking, in particular on pathways leading to type 2 diabetes, as well as on type 2 diabetes itself. The findings of this study could now be further tested by assembling data from randomised trials of smoking cessation, where postintervention data on measures of central adiposity are available. If confirmed, a tendency for smokers to acquire an ‘apple shape’ due to increasing central adiposity might provide a novel health promotion message to encourage smoking cessation, and appropriate new interventions should then be designed and evaluated as part of overall tobacco control policies in society.
